# Transcriptional progression during meiotic prophase I reveals sex-specific features and X chromosome dynamics in human fetal female germline

**DOI:** 10.1371/journal.pgen.1009773

**Published:** 2021-09-09

**Authors:** Xueying Fan, Ioannis Moustakas, Vanessa Torrens-Juaneda, Qijing Lei, Geert Hamer, Leoni A. Louwe, Gonneke S. K. Pilgram, Karoly Szuhai, Roberto Matorras, Cristina Eguizabal, Lucette van der Westerlaken, Hailiang Mei, Susana M. Chuva de Sousa Lopes

**Affiliations:** 1 Department of Anatomy and Embryology, Leiden University Medical Center, Leiden, The Netherlands; 2 Sequencing Analysis Support Core, Department of Biomedical Data Sciences, Leiden University Medical Center, Leiden, The Netherlands; 3 Center for Reproductive Medicine, Reproductive Biology Laboratory, Amsterdam Reproduction and Development Research Institute, Amsterdam University Medical Centers, Location AMC, Amsterdam, the Netherlands; 4 Department of Gynaecology, Leiden University Medical Center, Leiden, The Netherlands; 5 Department of Cell and Chemical Biology, Leiden University Medical Center, Leiden, The Netherlands; 6 IVIRMA, IVI Bilbao, Bilbao, Spain; Human Reproduction Unit, Cruces University Hospital, Bilbao, Spain; Department of Obstetrics and Gynecology, Basque Country University, Spain; Biocruces Bizkaia Health Research Institute, Bilbao, Spain; 7 Cell Therapy, Stem Cells and Tissues Group, Basque Centre for Blood Transfusion and Human Tissues, Galdakao, Spain; 8 Biocruces Bizkaia Health Research Institute, Cell Therapy, Stem Cells and Tissues Group, Barakaldo, Spain; 9 Department for Reproductive Medicine, Ghent University Hospital, Ghent, Belgium; Cornell University, UNITED STATES

## Abstract

During gametogenesis in mammals, meiosis ensures the production of haploid gametes. The timing and length of meiosis to produce female and male gametes differ considerably. In contrast to males, meiotic prophase I in females initiates during development. Hence, the knowledge regarding progression through meiotic prophase I is mainly focused on human male spermatogenesis and female oocyte maturation during adulthood. Therefore, it remains unclear how the different stages of meiotic prophase I between human oogenesis and spermatogenesis compare. Analysis of single-cell transcriptomics data from human fetal germ cells (FGC) allowed us to identify the molecular signatures of female meiotic prophase I stages leptotene, zygotene, pachytene and diplotene. We have compared those between male and female germ cells in similar stages of meiotic prophase I and revealed conserved and specific features between sexes. We identified not only key players involved in the process of meiosis, but also highlighted the molecular components that could be responsible for changes in cellular morphology that occur during this developmental period, when the female FGC acquire their typical (sex-specific) oocyte shape as well as sex-differences in the regulation of DNA methylation. Analysis of X-linked expression between sexes during meiotic prophase I suggested a transient X-linked enrichment during female pachytene, that contrasts with the meiotic sex chromosome inactivation in males. Our study of the events that take place during meiotic prophase I provide a better understanding not only of female meiosis during development, but also highlights biomarkers that can be used to study infertility and offers insights in germline sex dimorphism in humans.

## Author summary

Meiosis is a specialized and strictly-regulated cellular process and any genetic defects that emerge during meiotic prophase I may lead to the defective development of the gametes. These defects will result in either infertility or genetic diseases in the offspring, with significant consequences for the quality of life. We have limited understanding of the events that take place during human female meiotic prophase I, as it largely takes place during development. However, sequencing technologies are providing essential knowledge of this process. Here, we have analysed single-cell RNA sequencing data of human germ cells and identified the molecular signatures associated with progression through the different stages of the female meiotic prophase I. Interestingly, we observed that many genes differentially expressed during different female meiotic prophase I stages are related to male fertility. To further uncover sex-specific and conserved features, we have also compared female to male germ cells during meiotic prophase I. Our study fills an important gap regarding the events that take place during human female meiotic progression and highlights biomarkers that may prove important to develop infertility-associated disease models in vitro.

## Introduction

Meiosis is a particular cell division that aims to produce fully functional haploid gametes, necessary for the formation of a diploid zygote after fertilization [1]. Unlike male germ cells that enter meiosis only during puberty and complete the whole process within a period of two months (about 74 days in humans), female meiosis initiates during fetal development but only completes after fertilization, decades later. In contrast to mouse that shows a transient rostro-caudal wave during meiotic entry [2,3], human female fetal germ cell (FGC) development is strongly asynchronous during the second trimester [4–6]. By 18 weeks post-fertilization (WPF), corresponding to 20 weeks of gestation, distinct cellular states, showing characteristic morphology and expressing specific markers, are present in different compartments of the ovary, that is the outer cortex, germinal cords and inner cortex [5,7,8]. Human female DDX4 (or VASA) positive germ cells at diplotene are encapsulated in primordial follicles, whereas germ cells in earlier stages of prophase I are confined to the so-called germinal cords, that resemble seminiferous tubes in males [9]. The most peripheral germ cells, under the germinative epithelium, retain expression of pluripotency markers, such as POU5F1 (or OCT4) and NANOG [7,8].

During fetal development, female gem cells go through 4 stages of meiotic prophase I (leptotene, zygotene, pachytene and diplotene) and at birth the large majority is arrested in late diplotene (dictyate stage) (reviewed in [10]). Oocytes in dictyate stage have a large nucleus, also referred to as germinal vesicle (GV) and are individually encapsulated by one layer of squamous granulosa cells, in primordial follicles (reviewed in [11]). Oocytes can remain in dictyate for about 50 years and the last stage of prophase I (diakinesis) occurs prior to metaphase I (MI). During prophase I, the complex and precise process of genetic homologous recombination is orchestrated [12]. This involves several steps that include homologous chromosome alignment, pairing and synapsis, formation of a telomere bouquet, formation and disassembly of the synaptonemal complex, induction of DNA double-strand breaks (DSB) and DSB repair by homologous recombination leading to crossover and non-crossover events [12–14]. Aberrant homologous recombination is considered an important cause for aneuploidy, as crossover sites play an important role in correct chromosome alignment and segregation during MI [15]. In addition, mutations in genes related to the recombination process have been found responsible for infertility and premature ovarian insufficiency [16].

Differences in X chromosome dosage between sexes are important for sexual dimorphism and fertility [17]. In female (XX) somatic cells, one of the two X chromosomes is silenced (XaXi) to equalize the expression of X-linked genes with male (XY) cells, a process known as X chromosome inactivation (XCI); moreover, in both female and male somatic cells, the active X chromosome is overexpressed to balance X-linked expression with that of the autosome pairs, a process known as X chromosome upregulation (XCU) (reviewed in [18]). In the mouse female germline, the silent X chromosome (Xi or Barr body) is reactivated before meiotic entry [19,20]. By contrast in the mouse male germline, the X and Y chromosomes are silenced (XY body) during meiotic recombination, when the autosome homologous chromosomes are synapsed [meiotic sex chromosome inactivation (MSCI)] [21,22]. The molecular mechanisms and dynamics of X chromosome regulation are relatively well understood in mice, but remain rather obscure in humans, in particular in the female germline [23–26].

Several studies on human adult male gametogenesis, using single-cell transcriptomics, have characterized the different meiotic stages and highlighted important meiotic male factors [27–29]. To gain knowledge of meiotic factors that regulate prophase I in females, we analysed single-cell transcriptomics data from human fetal female gonads [5]. We identified the molecular signature of the different female prophase I stages during development and compared those to the corresponding prophase I stages in (adult) males. Moreover, we compared the expression of X-linked genes between sexes. We hypothesize that, despite common factors responsible for the nuclear progression through meiosis, female and male germ cells in meiotic prophase I display a pronounced dimorphism in transcriptional profiles and X-linked dynamics.

## Results

### Progression of human female fetal germ cells through meiotic prophase I

Using an online-available single-cell sequencing dataset (Smart-Seq2) from fetal human gonads ranging from 5-26WPF [5], we extracted 1435 female cells, of which 948 cells (from N = 16 donors) were retained after quality control for further analysis. Using a Seurat-based workflow [30], we identified 9 main clusters (CL) and visualized them in a two-dimensional plot using non-linear dimensionality reduction algorithm tSNE. The 9 clusters corresponded to 3 major cell types, (*WT1*+ *COL3A1*+) gonadal somatic cells, (*CD68*+ *CD4*+) immune cells and (*KIT*+ *DAZL*+) fetal germ cells (FGC) (S1A–S1C Fig), and cluster identities were in agreement with the previously-reported cell identities [5]. We noticed that the FGC clusters, and in particular CL7 containing *ZP3*+ oocytes, expressed a significantly higher number of genes (TPM>0) per single cell than the somatic clusters (CL0, CL5, CL8) (Welch two-sample t-test, p<2.2e-16) (S1D Fig) and this sole parameter may be useful as criterium to separate females FGC from somatic cells.

**Fig 1:**
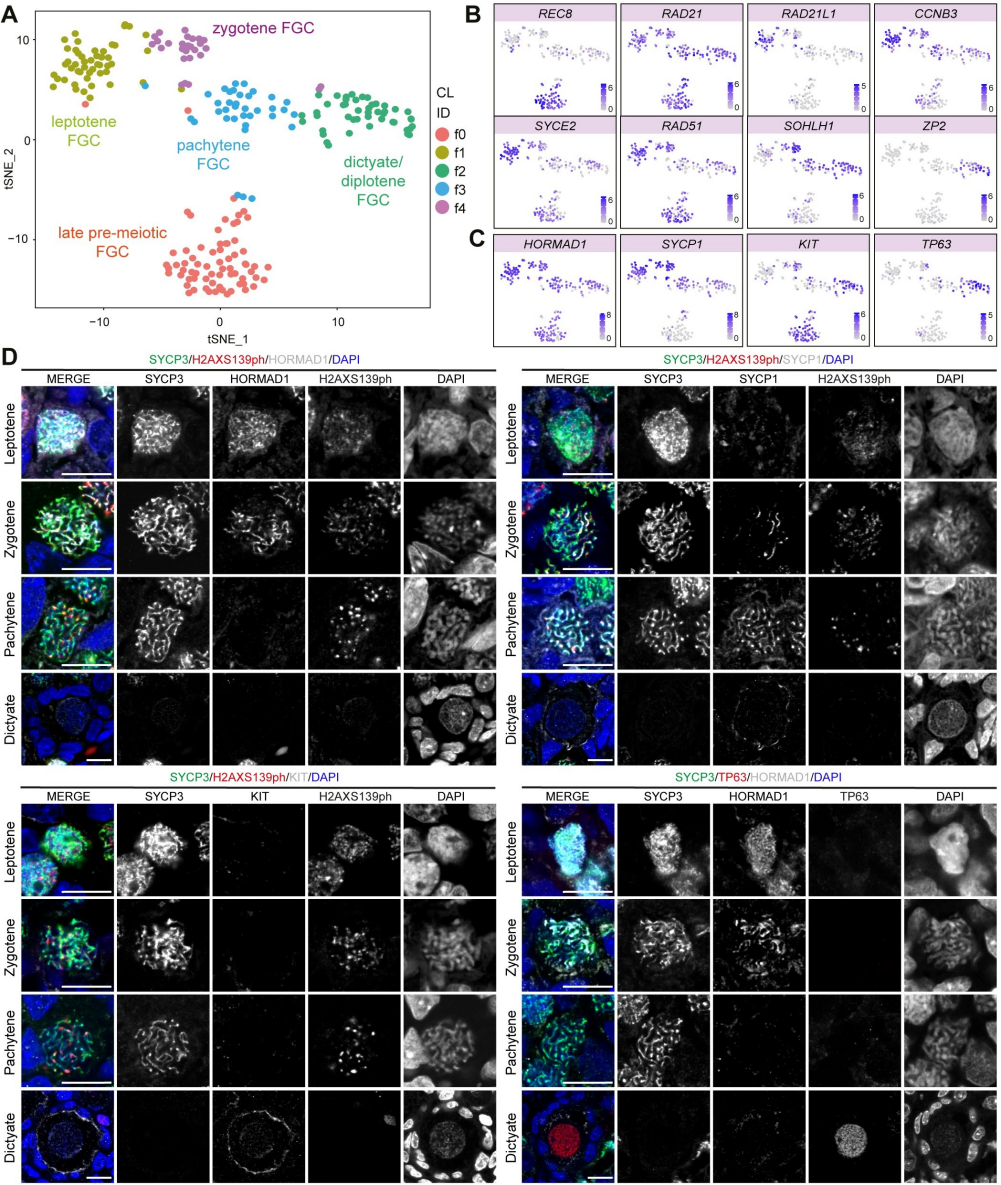
Human female fetal germ cells (FGC) during meiotic prophase I. **(A-C)** tSNE plots showing cell cluster identity (CL ID) in female FGC during meiotic prophase I **(A)** and depicting expression of known meiotic markers **(B-C)**. **(D)** Immunofluorescence of SYCP3, H2AXS139ph and selected fDEGs in leptotene, zygotene, pachytene and dictyate FGC in 14-18WPF ovarian sections. Scale bars are 10μm.

Next, we focused on (*STRA8*+) pre-meiotic late (CL6), (*SPO11*+) meiotic (CL3) and (*ZP3*+) oocytes (CL7) (S1 Fig) to extract the molecular signatures corresponding to the four different meiotic stages. We retained 227 cells from 11-26WPF and, using Seurat, we re-clustered the cells and obtained 5 sub-clusters (fCL) (Figs 1A and S2A) and the list of differentially expressed genes (fDEGs) for each fCL (pair-wise analysis, filtered for p_adjusted < 0.05, pct_1 > 0.6) (S1 Table). Analysis of several meiotic markers revealed that fCL0 corresponded to pre-meiotic (*REC8*+) FGC [31], fCL1 to (*SYCE2*+) FGC in leptotene [32], fCL4 to (*RAD51*+*SOHLH1*+) FGC in zygotene [33,34], fCL3 to (*SOHLH1*+) FGC in pachytene [33,34] and fCL2 to (*ZP2*+) oocytes in dictyate/diplotene [35] (Fig 1B). In agreement, pseudotime analysis (Monocle 3) also confirmed a trajectory from fCL0 (pre-meiotic FGC) to fCL2 (dictyate/diplotene FGC) (S2B and S2C Fig). Interestingly, FGC in pre-leptotene (or pre-meiotic S-phase) presenting high levels of cohesion complex protein *RAD21L1* and G_2_/M inhibiting cyclin *CCNB3*, and relatively lower levels of *RAD21* [36,37] seem to be included in fCL1 instead of fCL0 (Fig 1B).

To further validate each fCL, we first combined immunofluorescence for SYCP3 and H2AXS139ph (or γH2AX) with HORMAD1 [38] (Fig 1C and 1D). HORMAD1, a leptotene fDEG (S1 Table), showed strong accumulation on unsynapsed chromosome axes in leptotene FGC in which SYCP3+ axial elements of synaptonemal complex begin to form. Although *SYCP1* was highly expressed in leptotene and zygotene FGC (Fig 1C), SYCP1 [39] started to be detected in zygotene FGC, on the synapsed regions of homologous chromosomes and, in pachytene FGC, when SYCP1 largely colocalized with SYCP3 on the fully synapsed chromosomes that no longer showed HORMAD1 (Fig 1D). This is in line with male germ cell development, in which RNA expression often occurs before translation or activity of the corresponding protein [40]. Moreover, in agreement with previous studies [41,42], we confirmed that H2AXS139ph was still detected in female pachytene FGC (Fig 1D), suggesting that DSB repair in females is still occurring. By contrast, in male pachytene germ cells H2AXS139ph only marks the unsynapsed sex chromosomes [29]. In line with *KIT* and *TP63* expression (Fig 1C), both dictyate/diplotene fDEGs, KIT and TP63 were specifically expressed in dictyate oocytes in primordial follicles (Fig 1D).

### Dynamics of cytoskeleton-associated gene expression during female meiotic prophase I

**Fig 2:**
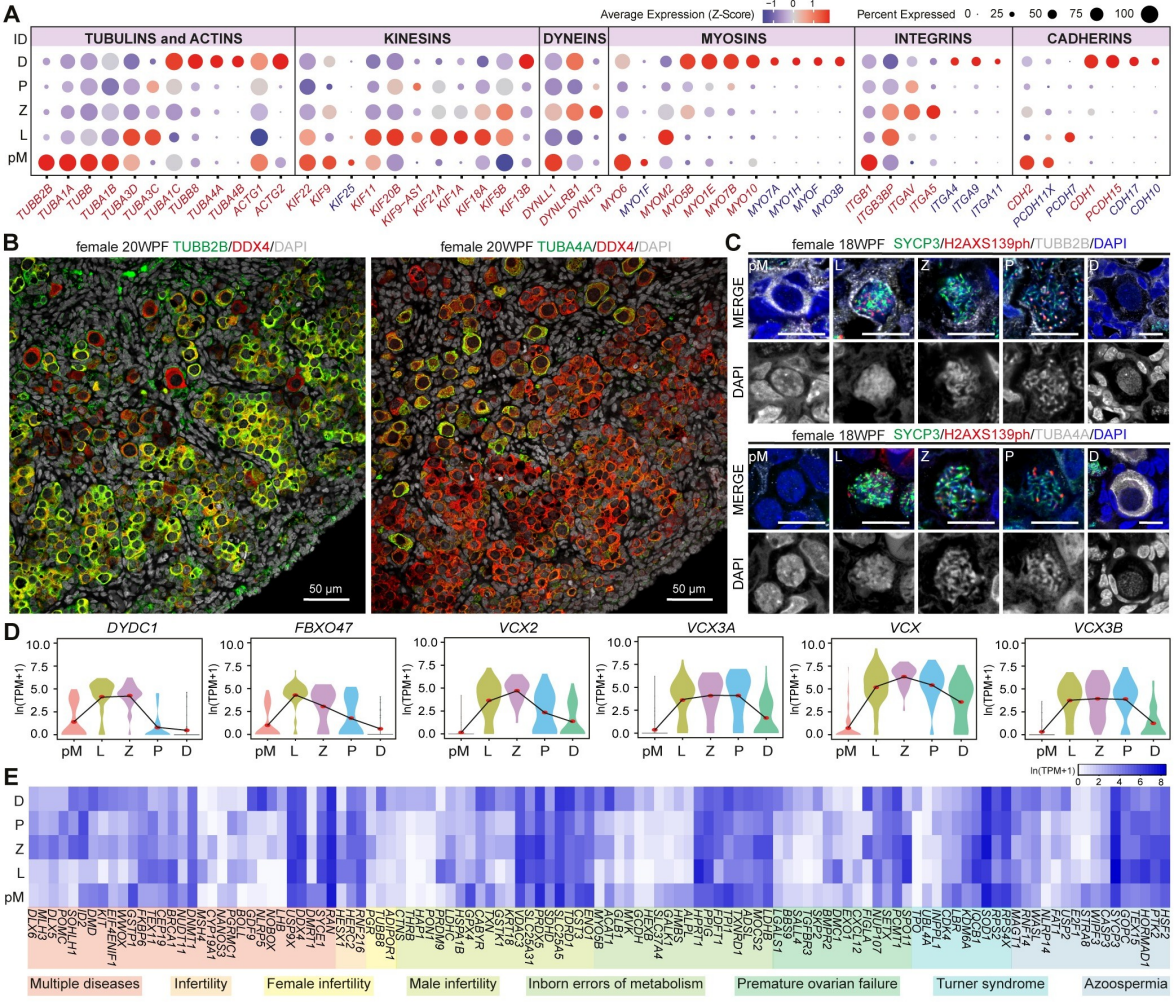
Expression of cytoskeletal and adhesion genes in female meiotic prophase I. **(A)** Dot plot showing scaled average expression (Z-score) of the cytoskeleton, motor and cell adhesion gene families in different meiotic prophase I stages (pre-meiotic, pM; leptotene, L; zygotene, Z; pachytene, P and dictyate/diplotene, D). Gene names in red are fDEGs and in blue are not fDEGs. **(B)** Immunofluorescence of 20WPF ovary for TUBB2B (left) and TUBA4A (right) with DDX4. Scale bars are 50μm. **(C)** Immunofluorescence of 18WPF ovary for TUBB2B (top) and TUBA4A (bottom) with SYCP3 and H2AXS139ph in selected FGC in pM, L, Z, P and D. Scale bars are 10μm. **(D)** Violin plots showing expression dynamics of selected fDEGs. Red dots mark mean expression per cluster. **(E)** Heatmap showing expression of fDEGs associated with reproductive-related disorders.

During meiotic prophase I, the chromosomes form crossovers followed by genetic recombination. Hence, it is expected that major changes occur in the composition of nuclear-associated factors. However, during meiotic prophase I, female FGC also undergo changes in cellular shape, from small FGC to large and round oocytes surrounded exclusively by granulosa cells. Therefore, we investigated the expression dynamics of cytoskeletal components during female meiotic prophase I. Meiotic fDEGs of the tubulin family (microtubules) included *TUBB*, *TUBB2B*, *TUBA1A* and *TUBA1B* in pre-meiotic FGC (fCL0), *TUBA3D* and *TUBA3C* in leptotene FGC (fCL1), and *TUBA1C*, *TUBA4*, *TUBA4B* and *TUBB8* in dictyate/diplotene FGC (fCL2) (Fig 2A). Using immunostaining, we confirmed a pronounced downregulation in TUBB2B from pre-meiotic to dictyate/diplotene FGC in 18-20WPF (Fig 2B and 2C) and oocytes in adult ovaries (S2D Fig). By contrast, TUBA4A showed a striking upregulation in dictyate/diplotene human FGC and oocytes in adult ovaries (Figs 2B, 2C and S2E). This dynamic pattern of expression of different tubulin subtypes may be associated and play different roles during the cytoplasmic transition from pre-meiotic FGC to oocyte, that occurs parallel to the nuclear progression throughout meiotic prophase I.

From the actin family (microfilaments), the gamma actins *ACTG1* and *ACTG2* were highly expressed specifically in pre-meiotic and diplotene FGC, respectively (Fig 2A). In addition, different members of the three major cytoskeletal motor proteins (kinesins, dyneins and myosins) as well as distinct isoforms of the integrin family (cell-extracellular matrix adhesion), cadherins (cytoskeletal-cell adhesion) and protocadherins (cell-cell adhesion) family were also differentially expressed at different stages of meiotic prophase I in human female FGC (Fig 2A). In addition to pronounced changes in the expression of cytoskeletal components, our results also revealed a clear shift in the expression of surface decoration components from pre-meiotic FGC to oocyte.

### Female meiotic fDEGs associate with diseases of the human reproductive system

Analysis of the meiotic fDEGs also revealed several genes not previously associated with meiotic prophase I in females, such as *DYDC1* and *FBXO47* that showed upregulation during leptotene (Fig 2D). By contrast, *Dydc1* has been reported to regulate acrosome biogenesis during mice spermatogenesis [43] and *Fbxo47* was shown to be specifically expressed in meiotic prophase I and gene knockout leads to male infertility in mice [44]. Moreover, we noticed that the VCX-family members *VCX*, *VCX2*, *VCX3A* and *VCX3B* were also fDEGs, up-regulated during meiotic prophase I (Fig 2D). The VCX-family are X chromosome-linked genes reported to be restricted to male germ cells [45,46]. Our analysis suggested that the VCX-family may also play a role during meiotic prophase I in the female germline in humans. Furthermore, comparing meiotic fDEGs with human disease-related genes, we observed that many meiotic fDEGs were associated with ‘female infertility’, ‘premature ovarian failure’, ‘Turner syndrome’ and ‘inborn errors of metabolism’, but notably many were also specifically associated with ‘male infertility’ and ‘azoospermia’ (Fig 2E), suggesting perhaps additional roles in male meiosis.

### Conserved molecular signature during meiotic prophase I progression between sexes

To compare the molecular signatures of the different stages of female meiotic prophase I to their male counterparts, we used an online-available single-cell transcriptomics dataset from the same platform (Smart-Seq2) and research group on human adult spermatogenesis [29]. First, quality control using similar parameters to the ones used for the female dataset was performed. From the 3244 cells, 2845 cells from 8 healthy male adult donors were retained. After performing unsupervised clustering analysis and visualization with t-SNE, 14 clusters (mCL) were detected (S3A and S3B Fig). Based on clustering annotation from Wang and colleagues [29] and known germline marker genes, we identified specific meiotic clusters of interest: (*SCML1*+) germ cells in leptotene (mCL3), (*TDRG1*+) germ cells in zygotene (mCL1), (*TDRG1+*, *OVOL2*+, *NME8*+) germ cells in early pachytene (mCL7), (*OVOL2*+, *NME8*+) germ cells in late pachytene (mCL5) and (*OVOL2*+, *NME8*+) germ cells in diplotene (mCL6) (S3C Fig). Next, we calculated the male DEGs (mDEGs) corresponding to each of the 14 clusters (S2 Table) (pair-wise analysis, filtered for p_adjusted < 0.05, pct_1 > 0.6). We investigated the expression of the cytoskeleton-associated genes in the obtained male clusters and observed that many fDEGs were not conserved in male germ cells during progression through meiotic prophase I (S3D Fig). As an example, in contrast to female FGC, neither TUBB2B nor TUBA4A were significantly expressed during male prophase I (S3D and S3E Fig). Our results contribute to identify key genes that may be involved in sex-specific morphological changes taking place during progression through female meiotic prophase I.

**Fig 3:**
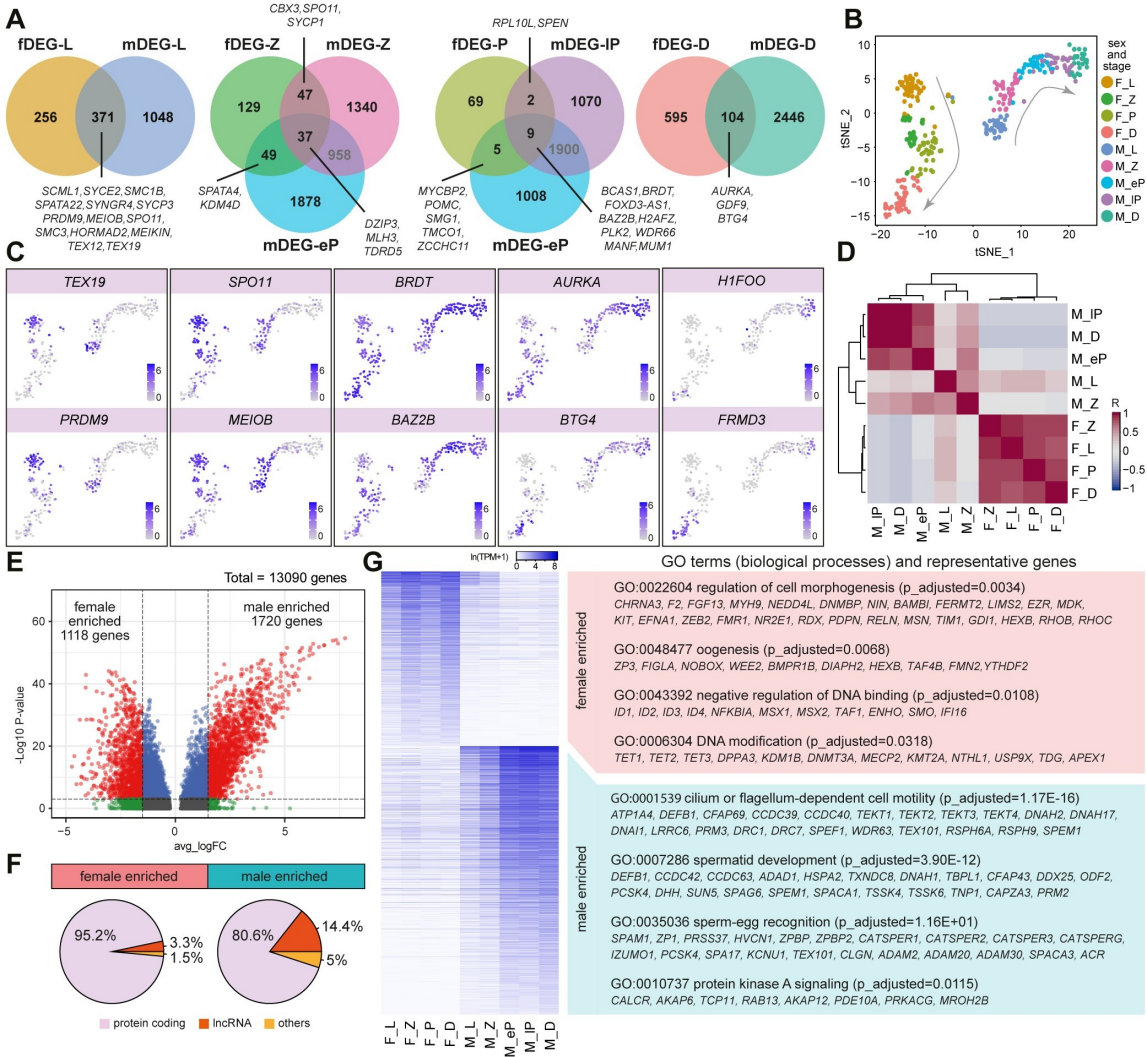
Meiotic differentially expressed genes (DEGs) between females and males. **(A)** Venn diagrams showing the intersection of fDEGs and mDEGs for each stage during meiotic prophase I (leptotene, L; zygotene, Z; early pachytene, eP; late pachytene, lP and dictyate/diplotene, D). **(B-C)** tSNE plots of female and male germ cells in meiotic prophase I coloured by sex and stage **(B)** and showing expression of selected genes/DEGs during meiotic prophase I **(C)**. F_L, female leptotene; F_Z, female zygotene; F_P, female pachytene; F_D, female dictyate/diplotene; M_L, male leptotene; M_Z, male zygotene; M_eP, male early-pachytene; M_lP, male late-pachytene; M_D, male diplotene. **(D)** Spearman correlation matrix across stages for both sexes during meiotic prophase I. Colour key is scaled by correlation coefficient R. **(E)** Volcano plot showing differentially expressed genes between meiotic female and male germ cells. **(F)** Pie chart showing the percentage of protein-coding, long-non coding (lnc) RNA and other biotypes for female or male enriched genes. **(G)** Heatmap showing expression of the differentially expressed genes obtained between meiotic female and male germ cells, together with representative GO terms (biological processes) and representative genes.

We intersected the meiotic stage-specific fDEGs with mDEGs counterparts (Fig 3A) and observed that many of the conserved DEGs between sexes in each stage were associated with meiosis-specific nuclear events, as opposed to the sex-specific morphological changes. For instance, *SPO11*, *TEX19*, *PRDM9*, *MEIOB* and *SPATA22* are involved in DSB [47,48], *SYCE2*, *SYCP1*, *SYCP3*, *TEX12* are members of the synaptonemal complex [10,49], *MLH3*, *BRDT* and *KDM4D* are responsible for crossover and DSB repair [50–52]. Surprisingly, some conserved DEGs have only been previously reported as important in one sex, such as *GDF9*, *BTG*4 and *H1FOO* identified as oocyte-specific genes [53]; and *POMC* and *WDR66* associated with sperm function [54,55]. Further investigations will clarify whether these genes could potentially carry out functions in the other sex during meiotic prophase I.

To provide a balanced visualization of gene expression in male and female germ cells during prophase I, we merged the 158 cells from female meiotic prophase I (fCL1, fCL2, fCL3, fCL4) with 1312 cells from male meiotic prophase I (mCL1, mCL3, mCL5, mCL6, mCL7) (S3F and S3G Fig). Subsequently, to eliminate the data size effect, we randomly selected 160 representative cells from the male dataset and integrated them with female cells (Figs 3B and S3G). We visualised the expression of several common meiotic genes or DEGs between the sexes, dynamically expressed during prophase I, such as *TEX19* and *PRDM9* (enriched in leptotene), *SPO11* and *MEIOB* (enriched in leptotene and zygotene), *BRDT* and *BAZ2B* (peak expression in pachytene), *AURKA* and *BTG4* (gradient from leptotene to diplotene), and *H1FOO* and *FRMD3* (enriched in diplotene) (Fig 3C). For each CL, we calculated the gene means, selected the top 100 most highly variably expressed genes and calculated the Spearman correlation matrix. We observed a principal separation according to sex, suggesting that sex differences drive gene expression during meiotic prophase I (Fig 3D).

### Divergent sex-enriched features during meiotic prophase I

To further characterise sex differences during meiotic prophase I, we calculated the DEGs between sexes (Fig 3E). We identified 1118 meiotic-female-enriched DEGs and 1720 meiotic-male-enriched DEGs (adjusted p-value <0.01 and absolute average log_e_ transformed fold change >1.5) (Fig 3E and S3 Table). First, we checked the RNA biotypes of these sex-specific meiotic-enriched DEGs and noticed that 14.4% of male DEGs were long non-coding RNA (lncRNA) in contrast to 3.3% in females (Fig 3F). Next, we performed a Gene Ontology (GO) enrichment analysis, that confirmed the pronounced sex-specific morphogenesis that takes place during progression through meiotic prophase I: meiotic-female-enriched GO terms included ‘cell morphogenesis’ and ‘oogenesis’ and meiotic-male-enriched GO terms included ‘cilium or flagellum-dependent cell motility’, ‘spermatid development’ and ‘sperm-egg recognition’ (Fig 3G and S4 Table). In addition, we observed that meiotic-female-enriched GO terms also included ‘regulation of DNA binding’ and ‘DNA modification’ (Fig 3G), suggesting that transcriptional regulation and epigenetic remodelling associated with regulation of DNA methylation (*TET1*, *TET2*, *TET3*, *DNMT3A)* diverge between sexes during meiotic prophase I.

### Regulation of DNA methylation during meiotic prophase I differs between sexes

**Fig 4:**
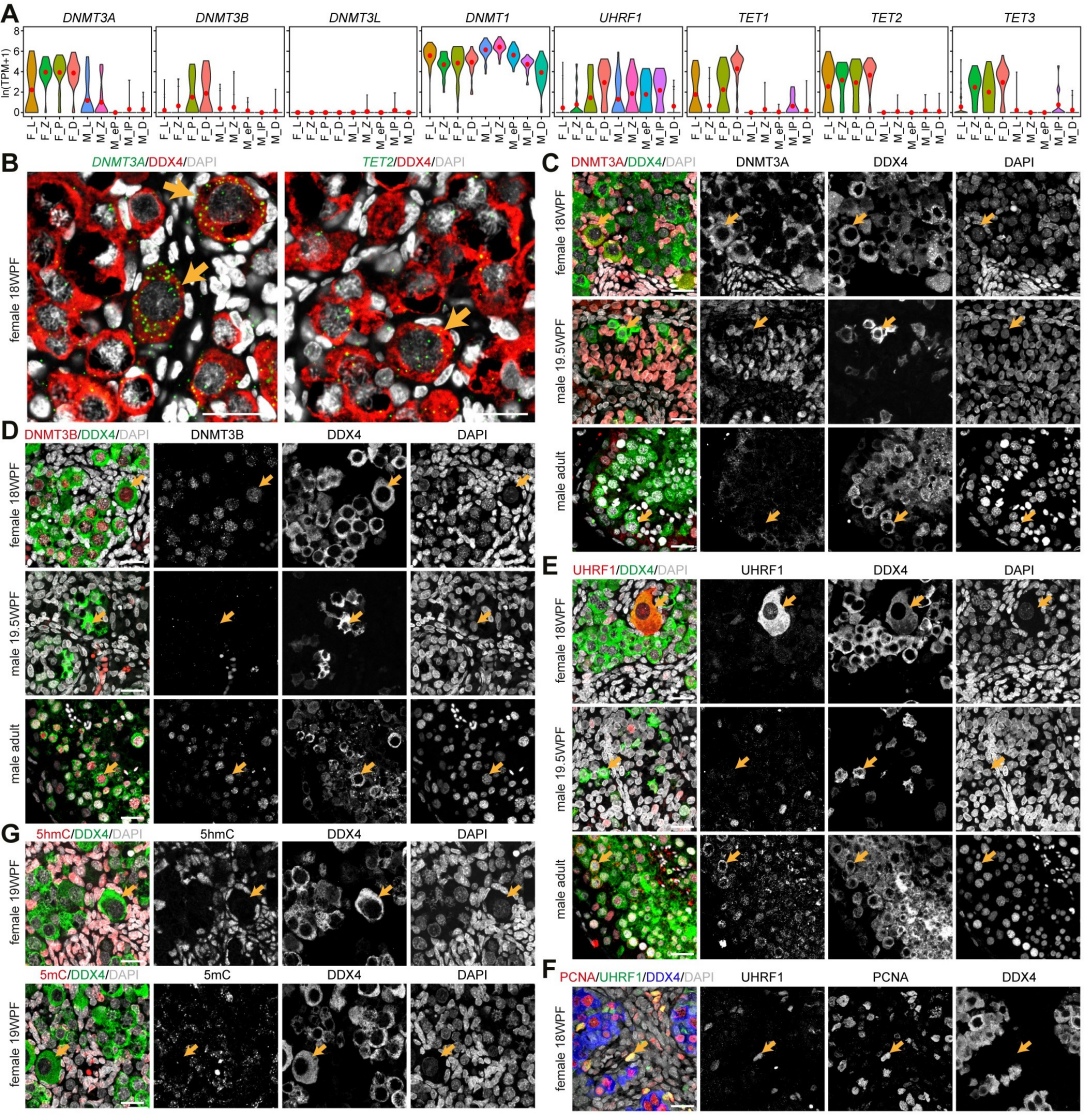
Regulation of DNA methylation during meiotic prophase I in males and females. **(A)** Violin plots showing expression of selected enzymes involved in the regulation of DNA methylation. Red dots mark mean expression per cluster. **(B)** RNA FISH of *DNMT3A* (left) or *TET2* (right) together with DDX4 in 18WPF ovaries. Orange arrows indicate dictyate/diplotene oocytes. **(C-E)** Immunofluorescence for DNMT3A **(C)**, DNMT3B **(D)** and UHRF1 **(E)** with DDX4 in 18WPF ovaries, 19.5WPF testes and adult testes. Orange arrows in 18WPF ovaries indicate dictyate/diplotene oocytes and in testes indicate representative DDX4+ germ cells. Scale bars are 20μm. **(F)** Immunofluorescence for UHRF1 and PCNA in 18WPF ovaries. Orange arrows indicate representative UHRF1+PCNA+ proliferating gonadal somatic cells. Scale bars are 20μm. **(G)** Immunofluorescence for 5-hydroxymethylcytosine (5hmC) and 5-methylcytosine (5mC) in 19WPF ovaries. Orange arrows indicate dictyate/diplotene oocytes. Scale bars are 20μm.

Violin plots for (co)enzymes involved in the regulation of DNA methylation suggested divergent expression dynamics between female and male germline during meiotic prophase I regarding *DNMT3A*, *DNMT3B*, *DNMT3L*, *DNMT1*, *UHRF1*, *TET1*, *TET2* and *TET3* (Fig 4A). Using RNA FISH, we confirmed strong localization of *DNMT3A* and *TET2*, particularly in dictyate/diplotene oocytes (18WFP) (Fig 4B). In agreement, cytoplasmic DNMT3A was expressed in female dictyate oocytes (Fig 4C), reminiscent of the cytoplasmic expression reported in human GV to metaphase II (MII) oocytes [56]. Furthermore, DNMT3A was absent from adult male germ cells, as those should already have acquired DNA methylation [57], but was also absent from both POU5F1+ and DDX4+ fetal male FGCs (19.5WFP) (Figs 4C and S4A), and from POU5F1+ fetal female FGCs (18WFP) (S4A Fig). In contrast to the transcriptomics data, we observed nuclear DNMT3B in both female and male meiotic germ cells (Fig 4D), but neither in POU5F1+ and DDX4+ fetal male FGC (Figs 4D and S4B), nor in POU5F1+ fetal female FGCs (18WFP) (S4B Fig). Next, we validated the strong sex differences in the expression of UHRF1. We report cytoplasmic localization in female dictyate/diplotene oocytes (Figs 4E and S4C), but similarly to what has been described in mice [58], we observed a pronounced translocation from the cytoplasm to the nucleus at the pachytene stage in adult male germ cells (Fig 4E). In addition, nuclear UHFR1 was also observed in PCNA+ proliferating female gonadal somatic cells (Fig 4F), but was absent from POU5F1+ and DDX4+ fetal male FGC (Figs 4E and S4D) and from POU5F1+ fetal female FGCs (18WFP) (S4D Fig).

Finally, we evaluated the levels of 5-hydroxymethylcytosine (5hmC) and 5-methylcytosine (5mC), but those were low/absent in all female and male FGC compared to the surrounding gonadal somatic cells (Figs 4G and S4E–S4H), but as expected 5mC was detected in male adult male germ cells (S4H Fig). Taken together, although the machinery that regulates DNA methylation seemed present in female FGC during second trimester, the re-establishment of DNA methylation may take place at a later stage.

### X-linked expression differs between sexes during meiotic prophase I

**Fig 5:**
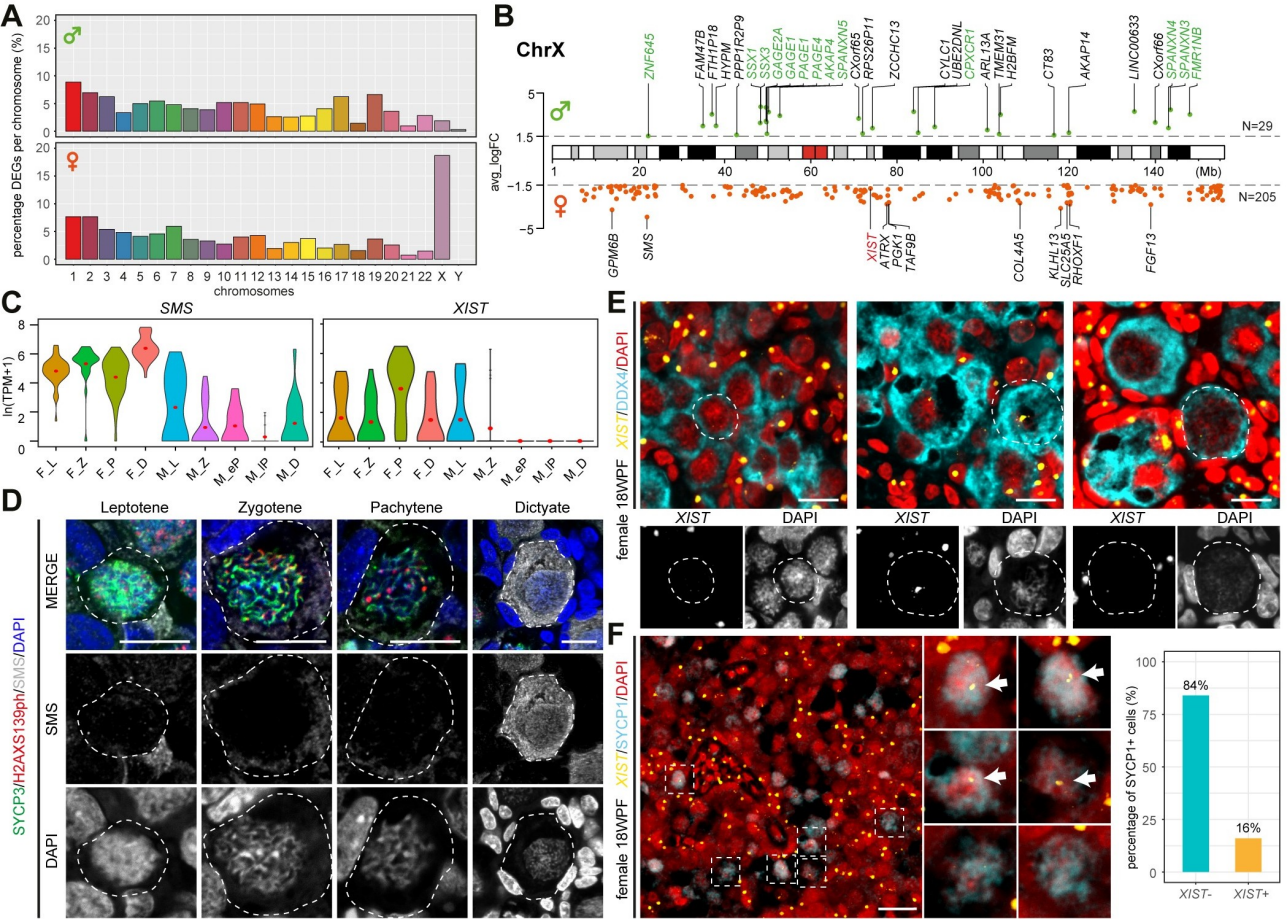
Differences in X-linked expression between the sexes. **(A)** Bar plot showing the percentage of meiotic male-DEGs (top) and female-DEGs (bottom) per chromosome. **(B)** Loci map of the X-linked meiotic male-DEGs (top) and female-DEGs (bottom) on the ChrX. Genes in green belong to the family of cancer/testis antigens, *XIST* is in red. **(C)** Violin plots showing expression of *SMS* and *XIST*. Red dots mark mean expression per cluster (pre-meiotic, pM; leptotene, L; zygotene, Z; pachytene, P and dictyate/diplotene, D). **(D)** Immunofluorescence for SMS, SYCP3 and H2AXS139ph in leptotene, zygotene, pachytene and dictyate FGC in 14-18WPF ovarian sections. Scale bars are 10μm. **(E)** RNA FISH for *XIST* combined with immunofluorescence for DDX4 in 18WFP ovaries. White dashed lines indicate selected DDX4+ FGC shown with separate channels for *XIST* and DAPI (bottom). Scale bars are 10μm. **(F)** RNA FISH for *XIST* combined with immunofluorescence for SYCP1 in 18WFP ovaries (left) and quantification of SYCP1+ FGC regarding *XIST* expression (right). White dashed boxes indicate selected SYCP1+ FGC, magnified in the middle panels. White arrows indicate *XIST*. Scale bars are 20μm.

When the sex-specific meiotic-DEGs were mapped to their chromosomal loci, we observed a comparable distribution, with the exception of the X chromosome [18.3% X-linked in females (205/1118 genes) versus 1.7% in males (29/1720 genes)] (Fig 5A and 5B and S3 Table). The X-linked female meiotic-DEGs showed expression throughout prophase I and consisted not only of X-linked genes that escape XCI (ChrX-E) (see [59] for list of ChrX-E genes), but also of X-linked genes that are subjected to XCI (ChrX-S) (S5A Fig). Although males only have one ChrX, we detected 29 X-linked male meiotic-DEGs, 13 belonging to the Cancer-Testis Antigen Gene (CTAG) family [60] (Figs 5B and S5A). We selected two X-linked female meiotic-DEGs for further validation, the protein-coding *SMS* and lncRNA *XIST* (Fig 5C). Using immunofluorescence, we confirmed high expression of SMS in particular in dictyate/diplotene oocytes (Fig 5D), compared to the lower expression in male germ cells in prophase I (S5B Fig).

**Fig 6:**
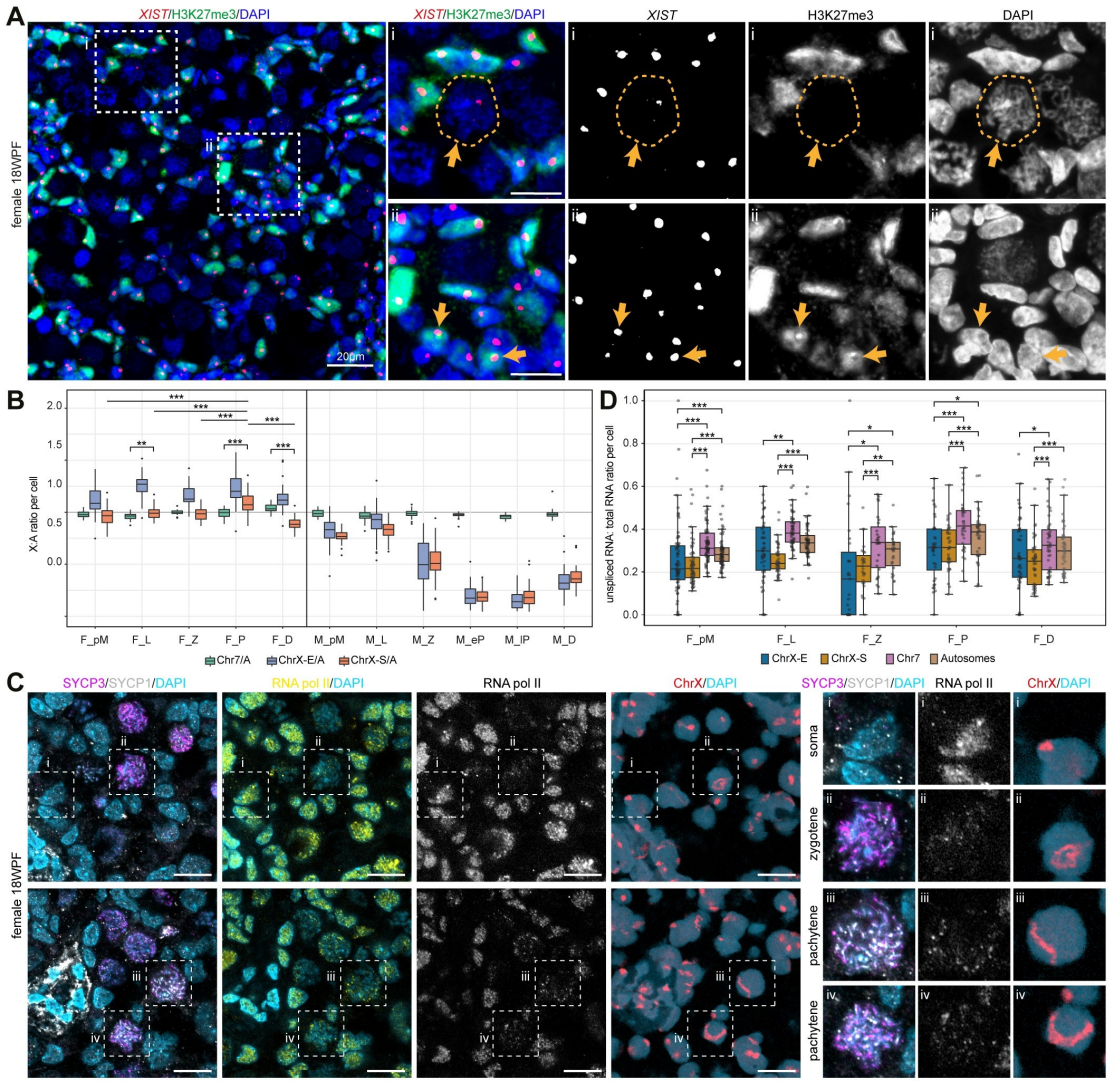
X-linked dynamics between sexes during meiotic prophase I. **(A)** RNA FISH for *XIST* combined with immunofluorescence for H3K27me3 in 18WFP ovaries. White dashed boxes indicate selected FGC, magnified on the right. i, orange arrows and dashed lines indicate a meiotic FGC showing *XIST* without H3K27me3; ii, orange arrows indicate somatic cells showing co-localization of *XIST* and H3K27me3. Scale bars are 20μm in the overview image (left) and 10μm in the high magnification images (right). **(B)** Box plot showing the ratio of mean expression of genes per cell from Chr7, ChrX-E and ChrX-S and mean expression of autosomes per cell. M-pM are male spermatogonial stem cells/spermatogonia cluster (mCL2); F_pM, female pre-meiotic germ cells; F_L, female leptotene; F_Z, female zygotene; F_P, female pachytene; F_D, female dictyate/diplotene; M_pM, male pre-meiotic germ cells; M_L, male leptotene; M_Z, male zygotene; M_eP, male early-pachytene; M_lP, male late-pachytene; M_D, male diplotene. Statistical significance was assessed using Wilcoxon rank-sum test or Wilcoxon signed-rank test for female germ cells, **P < 0.01, ***P < 0.001. **(C)** Immunofluorescence for SYCP3, SYCP1 and RNA pol II in 18WFP ovaries, sequentially used for DNA FISH of ChrX (image of the same region). Dashed boxes are showed in high magnification on the right. Scale bars are 10μm. **(D)** Box plot showing the ratio of unspliced RNA to total RNA for genes form Chr7, ChrX-E, ChrX-S and all autosomes per cell. Statistical significance was assessed using Wilcoxon signed-rank test for different groups in cells from each stage, *P < 0.05, **P < 0.01, ***P < 0.001.

Surprisingly, *XIST* was one of the female meiotic-DEGs upregulated during pachytene (Fig 5B and 5C). RNA FISH showed one strong *XIST* cloud, coating the inactive ChrX (Xi), in (DDX4-) gonadal somatic cells (Fig 5E); and although the majority of DDX4+ FGCs, including dictyate/diplotene oocytes, showed no *XIST*, some exhibited a clear *XIST* cloud (Fig 5E). Next, we investigated whether *XIST* was expressed in pachytene FGC by analysing the expression of *XIST* in high-expressing SYCP1+ FGC and observed that 16% of SYCP1+ FGC showed *XIST* accumulation (Fig 5F). No *XIST* expression was detected in male adult testicular cells (S5C Fig). To further study whether *XIST* initiated XCI in female pachytene FGC, we analysed the presence of H3K27me3 (marks Xi downstream of *XIST*). H3K27me3 colocalized with *XIST* in somatic cells, but was absent in female FGC (Figs 6A and S5D). Next, we investigated the expression levels of genes associated with XCI, including several X-linked genes (*JPX*, *FTX*, *RLIM*, *TSIX*, *XACT*, *KDM5C*), genes associated with XCI initiation and maintenance, and members of the polycomb repressive complex 1 (PRC1) and 2 (PRC2) [61,62] and some, such as *KDM5C*, *YY1*, *SPEN*, *RYBP* and *CBX8*, showed upregulation during pachytene stage (S5E Fig). Moreover, when a Pearson coefficient correlation was used to determine genes associated with *XIST* in pachytene FGC, among those positively correlated were *KDM5C*, *MGMT* and *SETDB1*, all previously associated with *XIST* regulation [63–65] (S5F Fig). However, these enzymes have a broad epigenetic function in the cell, suggesting that the upregulation of *XIST* in combination with the absence of H3K27me3 could reflect a global increase in transcriptional activity as observed between leptotene and (early) pachytene in males [40].

During the period of XCI, the Xa in mouse female FGC is overexpressed to approximate the expression of autosomes (A) and it remains overexpressed until the zygotene stage [18,66,67]. This phenomenon has been described in human female FGC [68], but the FGC were analysed by age and not stratified by stage of meiotic prophase I. We calculated the ChrX to autosome gene expression ratio (X:A) per cell using either only the dosage-sensitive ubiquitously-expressed X-linked genes (from [68]) (S5G Fig) or all X-linked genes (Fig 6B). As expected, X:A in male germ cells decreased dramatically from leptotene to pachytene, demonstrating MSCI (Figs 6B and S5G). In female FGC, X:A was significantly upregulated in pachytene compared to X:A in other stages and compared to 7:A in pachytene (two-sided Wilcoxon test, p<1.1e-05), suggesting some degree of XCU, with the highest level in pachytene FGC (Fig 6B).

To distinguish between increased X-linked transcription (XCU) and a lower turnover (due to lower translation or degradation) compared to autosomal genes, we investigated the localization of RNA polymerase II (RNA pol II). We detected RNA pol II in both SYCP3-/SYCP1- cells, presumably gonadal somatic cells and in SYCP3+SYCP1+ meiotic FGC (Fig 6C). To clarify whether there was an accumulation of RNA pol II on the ChrX in zygotene and pachytene FGC (suggestive of XCU), the immunostained sections were used for DNA FISH and the same location imaged. The match between the images did not reveal a particular accumulation of RNA pol II (on the ChrX) in zygotene and pachytene FGC (Fig 6C), suggesting that the observed increased X:A ratio results from lower X-linked turnover. However, due to the DNA FISH procedure, some (DAPI+) nuclei were lost or changed shape, making it unfeasible to directly quantify the RNA pol II occupancy on the ChrX. To further resolve this issue, we evaluated the ratio of unspliced to total RNA in the ChrX-S (and ChrX-E) compared to Chr7 and autosomes and we confirm a global increase in transcriptional activity between pre-meiotic and pachytene female FGC (Fig 6D). Importantly, we report a consistent and significantly lower ratio of unspliced to total RNA in the ChrX-S (and ChrX-E) compared to Chr7 or autosomes (Fig 6D). Together, our results suggest that the increased X-linked expression in female pachytene FGC did not result from increased X-linked transcription (XCU), but rather resulted from a lower turnover or higher stability of X-linked genes.

## Discussion

After female sex determination, FGC progress through meiotic prophase I (reviewed in [3,14]). As this occurs during development, much less is known about the molecular regulation of female meiosis in the human, in comparison to male meiosis which can be almost entirely studied using adult testis biopsies. It is only recently, with the emergence of single-cell technologies, that we are gaining comprehensive knowledge on the molecular identities of different cellular states during human gametogenesis [69] and comparing these between different species [28].

Through our systematic analysis and incorporation of male and female datasets, we uncovered the expression of several sex-specific genes that may assist in sexual dimorphism during meiotic prophase I. The extensive cytoplasmic reorganization and cytoskeletal dynamics that occur in parallel with nuclear rearrangements, during oocyte maturation (in adulthood) are well studied [70]. Here, we show that a dynamic expression of cytoskeletal components, such as tubulins, myosins and kinesis, also take place during female meiotic prophase I. Different tubulin isotypes, together with their associated post-translational modifications, known as the ‘tubulin code’, have direct impact on the properties and functionally of microtubules in the cell [71,72]. As an example, missense mutations in *TUBB8* have been identified as a cause of sterility in human females due to meiotic arrest [73] and in agreement we found high expression of *TUBB8* in diplotene oocytes. This suggests that regulation of cytoskeletal components, such as *TUBB8* during meiosis, could be crucial for oocyte development. This highlights the complexity of events occurring not only inside, but also outside the nucleus, such as organelle reorganization, increase in diameter and formation of cytoplasmic bridges between sister germ cells [74]. Our results also revealed extensive changes in expression of integrins and (proto)cadherins, reflecting different cell-cell, cell-cytoskeletal and cell-matrix affinity in germinal cords and primordial follicles. Progress in understanding the molecular interactions of germ cells with the gonadal niche and the extracellular matrix will facilitate the optimization of assays to investigate human gametogenesis [69].

Absence of DNA methylation related enzymes have been reported in female POU5F1+ FGC in the first trimester [75–77] and this remains in female POU5F1+ FGC in the second trimester. At the onset of meiosis, oocytes show absence of DNA methylation, in contrast to male germ cells that acquire male-specific DNA methylation before meiotic entry [78], resulting in sex-differences during meiotic recombination [79]. We observed expression of *de novo* DNA methyltransferase *DNMT3A* and *DNMT3B* during female meiotic prophase I, even though increase in the levels of DNA methylation only takes place after birth [78]. Expression of DNMT3B during human spermatogenesis has been previously reported [80] and we now report nuclear DNMT3B also during female meiotic prophase I. By contrast, we were unable to observe nuclear DNMT3A and UHRF1, but report strong cytoplasmic localization in dictyate/diplotene oocytes. Moreover, high expression of *TET1*, *TET2*, *TET3* during female prophase I, suggested ongoing 5-hydroxymethylation [81], perhaps to ensure DNA demethylation. In mice, *Tet1* has been shown to regulate gene expression during female meiosis [82)] and deletion of *Tet1* or *Tet1/Tet2* resulted in reduced female fertility [82,83]. Overall, our results suggest that DNA methylation regulators show pronounced sex differences.

Although the role of *XIST* in XCI in humans is still a matter of debate [24–26], *XIST* RNA has been reported in human female (pre-)meiotic FGC [6,75,84]. We and others have observed that pre-meiotic female FGC show transcriptional activity from both ChrX [6,84], however the analysis of X-dynamics during the different stages of prophase I has not been performed. In our study, we observed *XIST* upregulation in female pachytene FGC by transcriptomics analysis and RNA FISH. Unfortunately, none of the fetuses showed heterozygous single nucleotide polymorphisms (SNPs) in the *XIST* RNA; hence it was not possible to distinguish between biallelic expression from homozygous DNA and monoallelic expression from heterozygous DNA. Moreover, as during meiotic prophase I, the chromosomes pair and stretch (forming the meiotic bouquet) and loci are in close proximity, we were unable to determine whether the *XIST* localised to one or both *XIST* loci, although it was clear that it did not coat the entire (stretched) ChrX. This, together with the absence of H3K37me3 from the ChrX, suggests that there is no transient XCI during pachytene. However, this can only be excluded after comparing RNA expression with parental genomic heterozygous SNPs from each fetus as previously shown for pre-meiotic FGC [6] and the genomic information was not available in the dataset used here [5].

In males, an increase of transcriptional activity was observed between leptotone and (early) pachytene [40]. In agreement, we report an increasing trend in the ratio unspliced/total RNA during meiosis in female FGC. In addition, we observed an increased X:A ratio in pachytene female FGC, suggestive of XCU. However, the transcriptional activity was in fact lower in the ChrX compared to autosomes in female FGC. Hence, the observed upregulation of X:A ratio may be explained by lower turnover of X-linked genes (less degradation or translation). In this respect, it has been reported that X-linked transcripts in both human and mouse indeed have longer half-lives than autosomal transcripts, leading to increased stability [85].

Providing a glimpse on the complex regulation of X-linked expression during prophase I in males, we showed evidence of a specific class of X-linked genes, the CTAG family, that is male-enriched during meiotic prophase I, during X-linked silencing occurring at MSCI. Notably, abnormal expression of CTAG family in males causes meiotic arrest and subsequent infertility [86–88].

In conclusion, through our systematic analysis and incorporation of male and female datasets, we highlight the molecular progression and X-linked dynamics during meiotic prophase I in female FGC and identified both sex-enriched and conserved features that may prove informative to target aneuploidy and infertility as well as to develop disease models for human gametogenesis.

## Materials and methods

### Ethics statement regarding the use of human tissue

Human gonads from elective abortion (without medical indication) were donated for research purposes with signed informed consent. The gestational age was established prior to the procedure by obstetric ultrasonography. The collection and use of fetal tissues were approved by the Medical Ethical Committee of the Leiden University Medical Centre (P08.087).

Human adult ovaries used were from diseased cancer patients, that had one ovary removed and cryopreserved for fertility preservation purposes. The permission to use the adult ovarian material for research purposes was obtained by signing informed consent. The research using adult ovarian material was approved by the Medical Ethical Committee of the Leiden University Medical Centre (CME 05/03K/YR).

Human adult testicular biopsies were obtained from 3 males aged 36–47, undergoing testicular biopsy in search of spermatozoa to perform an intracytoplasmic sperm injection (ICSI) cycle. Two of the males had a previous vasectomy and the other was diagnosed with idiopathic azoospermia. The karyotype as well levels of follicle-stimulating hormone, luteinizing hormone and testosterone were normal. In all cases, motile spermatozoa were retrieved in the biopsy. An aliquot of the sample was employed for this research project. The participants signed the informed consent, approved by the Basque Ethics Committee for Clinical Research (CEIC-E PI2014205).

### Immunofluorescence and imaging

Fetal gonads (female 14-20WPF and male 14-20WPF), adult ovarian cortex and adult testis biopsies were fixed overnight in 4% paraformaldehyde (PFA) at 4°C, and embedded in paraffin. Paraffin sections (5μm thickness) were deparaffinized and rehydrated using xylene, ethanol with sequential dilution (100%, 100%, 90%, 80%, 70%) and distilled water. Followed by 15 minutes (min) of antigen retrieval performed by heating the sections (98°C) submerged in 0.01M sodium citrate buffer (pH 6.0) or Tris-EDTA buffer (10mM Tris Base, 1mM EDTA, pH9.0) using a microwave (TissueWave 2, Thermo Scientific). The samples were allowed to cool down, followed by three times rinsing with PBS and 1 hour (h) blocking at room temperature (RT) in blocking buffer (5% BSA, 0.3% Triton X-100 in PBS). Sections were incubated with primary antibodies diluted in 1% BSA-0.05% Tween 20-PBS buffer at RT overnight and subsequently incubated with secondary antibodies for 1h at RT. The primary antibodies used were goat anti-SYCP3 (1:500, AF3750, R&D), mouse anti-phospho-Histone H2A.X (ser139) (1:1000, 05–636, Upstate), rabbit anti-KIT (1:200, A450229-2, DAKO), mouse anti-TP63 (1:100, ab735, Abcam), rabbit anti-HORMAD1 (1:400, ab155176, Abcam), rabbit anti-SYCP1 (ab15090, Abcam), goat anti-DDX4 (1:500, AF2030, R&D), rabbit anti-TUBB2B (1:100, abx026514, Abbexa), rabbit anti-TUBA4A (1:100, NBP2-67148, Novus Biologicals), mouse anti-DNMT3A (1:100, sc-373905, Santa Cruz), mouse anti-DNMT3B (1:100, IMG-184A, Imgenex), rabbit anti-5hmC (1:500, 39792, Active Motif), rabbit anti-UHRF1 (1:100, GTX113963, GeneTex), mouse anti-5mC (1:200, ab10805, Abcam), rabbit anti-SMS (1:200, HPA029849, Sigma Aldrich), goat anti-POU5F1 (1:200, sc-8628, Santa Cruz), mouse anti-PCNA (1:50, sc-56, Santa Cruz), rabbit anti-H3K27me3 (1:500, 07–449, Sigma Aldrich), and mouse anti-RNA Polymerase II RPB1 (1:50, 664906, BioLegend). The secondary antibodies used were Alexa Fluor 488 donkey anti-rabbit IgG (1:500, A-21206, Life Technologies), Alexa Fluor 594 donkey anti-mouse IgG (1:500, A-21203, Life Technologies) and Alexa Fluor 647 donkey anti-goat IgG (1,500, A-21447, Life Technologies). Nuclei were stained with 4′,6-diamidino-2-phenylindole (DAPI, Life Technologies) and ProLong Gold (Life Technologies) was used to mount the sections. Immunostained sections were imaged on an inverted confocal microscope (SP5 CLSM, Leica) with LAS software (Leica). ImageJ software was used for image analysis [89].

### RNA FISH

RNA FISH performed on paraffin sections (5μm thickness) using RNAscope Multiplex fluorescent reagent kit v2 (323100, Advanced Cell Diagnostics), following manufacturer instructions. Briefly, after deparaffinization, sections were pre-treated with H_2_O_2_, incubated in retrieval solution for 15min and treated with Protease Plus for 15min at 40°C in a HybEZ II oven (321720, Advanced Cell Diagnostics). Next, the probe mix was added to the sections and incubated 2h at 40°C. The probes used were RNAscope Probe-Hs-DNMT3A (419441-C1), RNAscope Probe-Hs-TET2 (420051-C1), RNAscope Probe-Hs-XIST-C2 (311231-C2). Probes from different channels (C1 or C2) were mixed by pipetting 1 volume of C2 probe to 50 volumes of C1 probe. After hybridization, the mRNA signal was amplified sequentially for each channel. Fluorophores used to detect signals were Opal 520 (1:1000, FP1487001KT, Akoya Biosciences) and Opal 570 (1:2000, FP1488001KT, Akoya Biosciences). Nuclei were stained with DAPI and ProLong Gold (Life Technologies) was used to mount the slides.

To combine immunofluorescence with RNA FISH, the sections were first used for RNA FISH hybridization with the probe mix and signal amplification as above. Thereafter, the sections were blocked with 10% normal horse serum (S-2000, Vector Laboratories) or normal swine serum (014-000-121, Jackson ImmunoResearch) in blocking buffer (1% BSA, 0.05% Tween-20 in PBS) overnight at 4°C. Sections were treated with primary antibodies for 2h at RT. HRP-linked donkey anti-goat IgG (1:500, 705-035-003, Jackson ImmunoResearch) and HRP-linked swine anti-rabbit Immunoglobulins (1:200, P0217, DAKO) were used as secondary antibodies for goat anti-DDX4 (1:500, AF2030, R&D) and rabbit anti-H3K27me3 (1:500, 07–449, Sigma Aldrich), rabbit anti-SYCP1 (ab15090, Abcam), respectively. After 30min at RT with secondary antibodies, sections were incubated with Opal 690 (1:800, FP1497001KT, Akoya Biosciences) for 10 min, counterstained with DAPI and mounted with ProLong Gold (Life Technologies). The sections were imaged on an inverted SP5 CLSM confocal microscope (Leica).

### DNA FISH

To combine immunofluorescence with DNA FISH, paraffin sections were first used for immunofluorescence for SYCP3, SYCP1 and RNA Polymerase II as above, but the slides were then mounted with CitiFluor Non-Hardening Antifadent (AF1/DAPI-15, CitiFluor) and scanned using a ZEISS LSM 900 Airyscan (Zeiss, Germany) or Pannoramic slide scanner P250 (3DHistech, Hungary). Thereafter, the coverslip was removed by immersing in PBS for 10min at RT. The slides were then incubated with 0.1% pepsin (pepsin from porcine gastric mucosa, P7000-100G, Sigma-Aldrich, St Louis, MO, USA) in 0.02M HCl for 15min at 37°C. To detect human ChrX, 100ng whole chromosome painting probe for the ChrX chromosome was hybridised per section. The probe labelling and hybridization reaction were performed as described [90,91]. The slides were scanned again after DNA FISH and the same region in the scanned section for immunofluorescence and DNA FISH were used for analysis.

### Analysis of single-cell RNA-seq dataset

The single-cell transcriptomic dataset from human fetal female gonads (TPM-normalized count tables; the embryo of 14WPF was removed due to unclear labelling) was obtained from Li and colleagues (accession number GSE86146) [5] and from human adult testes (TPM-normalized count tables) was obtained from Wang and colleagues (accession number GSE106487) [29]. For quality control of the female dataset, cells with a number of transcripts between 100,000 and 1,500,000 and a number of expressed genes above 2000 were kept for further analysis. For the male dataset, cells with a number of transcripts above 10000 and a number of expressed genes above 2000 were kept for further analysis. Next, an R workflow based on the package Seurat (v3.0.2) [30] was applied. Differentially expressed genes (DEGs) for each cell cluster was calculated using function “FindAllMarkers” in the Seurat workflow, followed by filtering for p_val_adj < 0.05 and pct_1 > 0.6. Gene chromosome name and Entrez ID were added using biomaRt (v2.42.1) [92] and "hsapiens_gene_ensembl" as dataset (Ensembl v100, GRCh38).

To integrate the female and male datasets containing meiotic cells, 158 fetal female cells from fCL1-fCL4 and 1312 adult male cells from mCL1, mCL3, mCL5-mCL7 were pooled. To balance the two datasets, we randomly selected 160 male representative cells from the merged dataset. In total, 318 cells were used for analysis. To integrate the female and male datasets containing meiotic and pre-meiotic cells, 227 fetal female cells from fCL0-fCL4 and 1595 adult male cells from mCL1-mCL3 and mCL5-mCL7 were pooled. To balance the two datasets, we randomly selected 230 male representative cells from the merged dataset. In total, 457 cells were used for analysis.

To calculate the correlation matrix between the different germ cells CL, the mean expression of all genes was calculated and the 100 most variable gene means were selected using R function rowVars, from package genefilter (v1.68.0). The “cor” function in R was used to compute the Spearman correlation coefficient. This matrix was used to generate a heatmap using function heatmap.2 from R package gplots (v3.0.4) and R function hclust was used with agglomeration method set to ‘complete’. The “cor.test” function in R was used to identify genes that correlated (Pearson correlation) with *XIST* expression in female pachytene FGC. Both the correlation coefficient and the significance level (p-value) of the correlation were obtained.

Gene ontology (GO) analysis for female and male meiotic-DEGs was performed by using “enrichGO” function from the DOSE package (v3.14.0) [93]. GO terms were called specifically for “biological process”.

X-linked genes that escape XCI (ChrX-E) were selected according to Balaton and colleagues [59]: 124 X-linked genes annotated as “PAR” (pseudoautosomal region), “E” (escape), “mostly E” (mostly escape), “mostly VE” (mostly variable escape) and “VE” (variable escape). The list of ubiquitously-expressed X-linked genes was selected according to Sangrithi and colleagues [68]. The mean expression of genes per cell from Chr7, ChrX (ChrX-S and ChrX-E) and autosomes (neither X-linked nor Y-linked genes) and ratio per cell of chromosome/autosome were calculated.

### Unspliced and spliced RNA analysis

Raw data (FASTQ files) were downloaded from the GEO, accession number GSE86146. First, the reads were aligned to the GRCh38 human genome using STARsolo (v2.7.3a) [94]. STARsolo was run with cell barcode and UMI-aware settings. A custom Python script was used for demultiplexing to create one BAM file per cell.⁠ UMI-tools (v1.0.1) [95] were used to remove the UMI duplicates. Next, per gene counts of spliced and unspliced molecules were counted by running velocyto [96]. The output of velocyto (a loom file) was processed with a Python script using the following packages: numpy (v1.19.5), scanpy (v1.7.2), pandas (v1.1.5), scvelo (v0.2.3), seaborn (v0.11.1), scipy (v1.5.3).

### Cell pseudotime and trajectory analysis

Monocle 3 (v0.2.1) was used to order the cells and generate the trajectory. In this workflow, we used Uniform Manifold Approximation and Projection (UMAP) for dimension reduction and parameters (num_dim = 7, n_neighbors = 6, min_dist = 0.15) were selected. The beginning of pseudotime was selected on the UMAP plot based on the position where the pre-meiotic FGC clustered.

### Association with disease-related genes

From DisGeNET v6.0 [97], the genes associated with disease-terms “azoospermia, C0004509”, “male infertility, C0021364”, “female infertility, C0021361”, “infertility, C0021359”, “ovarian failure, premature C0085215”, “Turner Syndrome, C0041408” and “inborn errors of metabolism, C0025521” were extracted (downloaded in April, 2020). The list of female mDEGs was intersected with the gene list from each disease-term. Genes belonging to several of the above categories were assigned “multiple diseases”.

### Quantification and statistical analysis

The Welch two-sample t-test was used to compare the number of genes expressed in female germ cells versus somatic cells, using R. The Wilcoxon rank-sum test (two-sided) was used to calculate pair-wise differences of ChrX-S/A for female FGC in different stages, using R. The Wilcoxon signed rank test (paired = TRUE) was used to calculate differences between ChrX-S/A and Chr7/A for female FGC and unspliced to total RNA ratio differences between chromosomes in each meiotic stage, using R.

## Supporting information

S1 Fig Major molecular states in female gonads during development. **(A-C)** tSNE plots showing cell cluster identity (CL ID) for female fetal gonadal cells (FGC, fetal germ cells) **(A)**, age in weeks post-fertilization (WPF) **(B)** and expression of markers for each major cell type **(C)**. **(D)** Box plot and cell density graph showing numbers of genes expressed per cell in the germline and somatic cells in the female gonads per cluster.
(TIF)

S2 Fig Characteristics of human female FGC during meiotic prophase I. **(A)** tSNE plot of female FGC in pre-meiotic and meiotic stages coloured by age in weeks post-fertilization (WPF). **(B-C)** Pseudotime analysis of female pre-meiotic and meiotic FGC by Monocle 3. UMAP plots show pseudotime **(B)** and cluster identification **(C)**. **(D)** Immunofluorescence for TUBB2B and DDX4 in primordial follicles with oocytes arrested in diplotene in adult ovary. Scale bars are 50μm in the overview image (left) and 10μm in the high magnification image (right). **(E)** Immunofluorescence for TUBA4A and DDX4 in primordial follicles with oocytes arrested in diplotene in adult ovary. Scale bars are 50μm in the overview image (left) and 10μm in the high magnification image (right).
(TIF)

S3 Fig Major molecular states in human adult testicular cells. **(A-C)** tSNE plots showing cell cluster identity (CL ID) representing the main cell types **(A)**, individual donors **(B)** and expression of known markers for each major cell population **(C)**. (SSC/SPG, spermatogonial stem cells/spermatogonia; L, leptotene; Z, zygotene; eP, early pachytene; lP, late pachytene; D, diplotene; SPC7, spermatocyte 7; S1/S2/S3/S4, four stages of spermatids). **(D)** Dot plot showing scaled average expression (Z-score) of cytoskeleton, motor and cell adhesion gene families in different meiotic prophase I stages. Gene names in red are mDEGs, in red and bold are also fDEGs and in blue are not mDEGs. **(E)** Immunofluorescence of TUBB2B, SYCP3 and H2AXS139ph (top) and TUBA4A, SYCP3 and H2AXS139ph (bottom) in adult testes. Orange arrows indicate TUBB2B or TUBA4A positive spermatids, orange dashed lines mark the seminiferous tubules. Scale bars are 20μm. **(F-G)** tSNE plots displaying female and male cells in L, Z, P and D, coloured by sex **(F)** and showing the selected male cells used in further analysis **(G)**.
(TIF)

S4 Fig Expression of DNA methylation regulators in human germ cells. **(A-B)** Immunofluorescence for DNMT3A **(A)** and DNMT3B **(B)** in POU5F1+ FGC in second trimester ovaries and testes. Orange arrows indicate representative POU5F1+ FGC. Scale bars are 20μm. **(C-D)** Immunofluorescence for UHRF1 in DDX4+ FGC **(C)** and POU5F1+ FGC **(D)** in second trimester ovaries and testes. Orange arrows indicate representative FGC. Scale bars are 20μm. **(E)** Immunofluorescence for 5hmC in POU5F1+ FGC in second trimester ovaries and testes. Orange arrows indicate representative POU5F1+ FGC. Scale bars are 20μm. **(F)** Immunofluorescence of 5hmC in DDX4+ FGC from second trimester and adult testes. Orange arrows indicate representative DDX4+ FGC. Scale bars are 20μm. **(G)** Immunofluorescence for 5mC in POU5F1+ FGC in second trimester ovaries and testes. Orange arrows indicate representative POU5F1+ FGC. Scale bars are 20μm. **(H)** Immunofluorescence of 5mC in DDX4+ FGC from second trimester and adult testes. Orange arrows indicate representative DDX4+ FGC. Scale bars are 20μm.
(TIF)

S5 Fig Aspects of X-linked dynamics during meiotic prophase I. **(A)** Heatmap of X-linked sex-enriched genes (ChrX-Escape and ChrX-Subject to XCI) in male and female germ cells during meiotic prophase I. **(B)** Immunofluorescence for SMS, SYCP3 and H2AXS139ph in adult testes. Orange arrowheads indicate SMS in spermatogonia, orange dashed lines mark the border of seminiferous tubule. Scale bar is 20μm. **(C)** RNA FISH for *XIST* combined with immunofluorescence for DDX4 in adult testes. White dashed box is shown in high magnification (bottom). Orange dashed lines mark the border of seminiferous tubule. Scale bars are 20μm. **(D)** Immunofluorescence for H3K27me3, SYCP3 and H2AXS139ph in 18WPF ovaries. Orange dashed boxes indicate the areas shown in high magnification. Orange arrows indicate somatic cells or FGCs in their meiotic prophase I stages. (soma, somatic cells; L/Z, late leptotene-early zygotene; Z, zygotene; P, pachytene). Scale bar is 20μm. **(E)** Heatmap showing expression of *XIST* and XCI related genes in female FGC during meiotic prophase I (pre-meiotic, pM; leptotene, L; zygotene, Z; pachytene, P and diplotene/dictyate, D). **(F)** Bar chart showing genes that showed correlation with *XIST* expression (Pearson correlation coefficient R>0.5 or R<-0.5) in female pachytene FGC. Colour key is scaled by adjusted P-value. **(G)** X:A ratio of female and male germ cells in different stages. Box plot shows the mean expression ratios of ubiquitously expressed genes (ubi) from Chr7, ChrX-E or ChrX-S to all ubiquitously expressed genes from autosomes per cell. Cells from M-pL were actually cells from male spermatogonial stem cells/spermatogonia cluster (mCL2). Statistical significance was assessed using Wilcoxon rank-sum test or Wilcoxon signed-rank test for female germ cells, **P < 0.01, ***P < 0.001.
(TIF)

S1 Table Differentially expressed genes during human female meiotic prophase I. (XLS)

S2 Table Differentially expressed genes in human adult testes. (XLS)

S3 Table Differentially expressed genes between male and female meiotic prophase I. (XLS)

S4 Table GO terms of female and male enriched genes. (XLS)
