# Supplementary figures and images for: Transcriptional progression during meiotic prophase I reveals sex-specific features and X chromosome dynamics in human fetal female germline
Source: PLoS Genet. 2021 Sep 9;17(9):e1009773. doi: 10.1371/journal.pgen.1009773 (PMC8428764; doi:10.1371/journal.pgen.1009773)

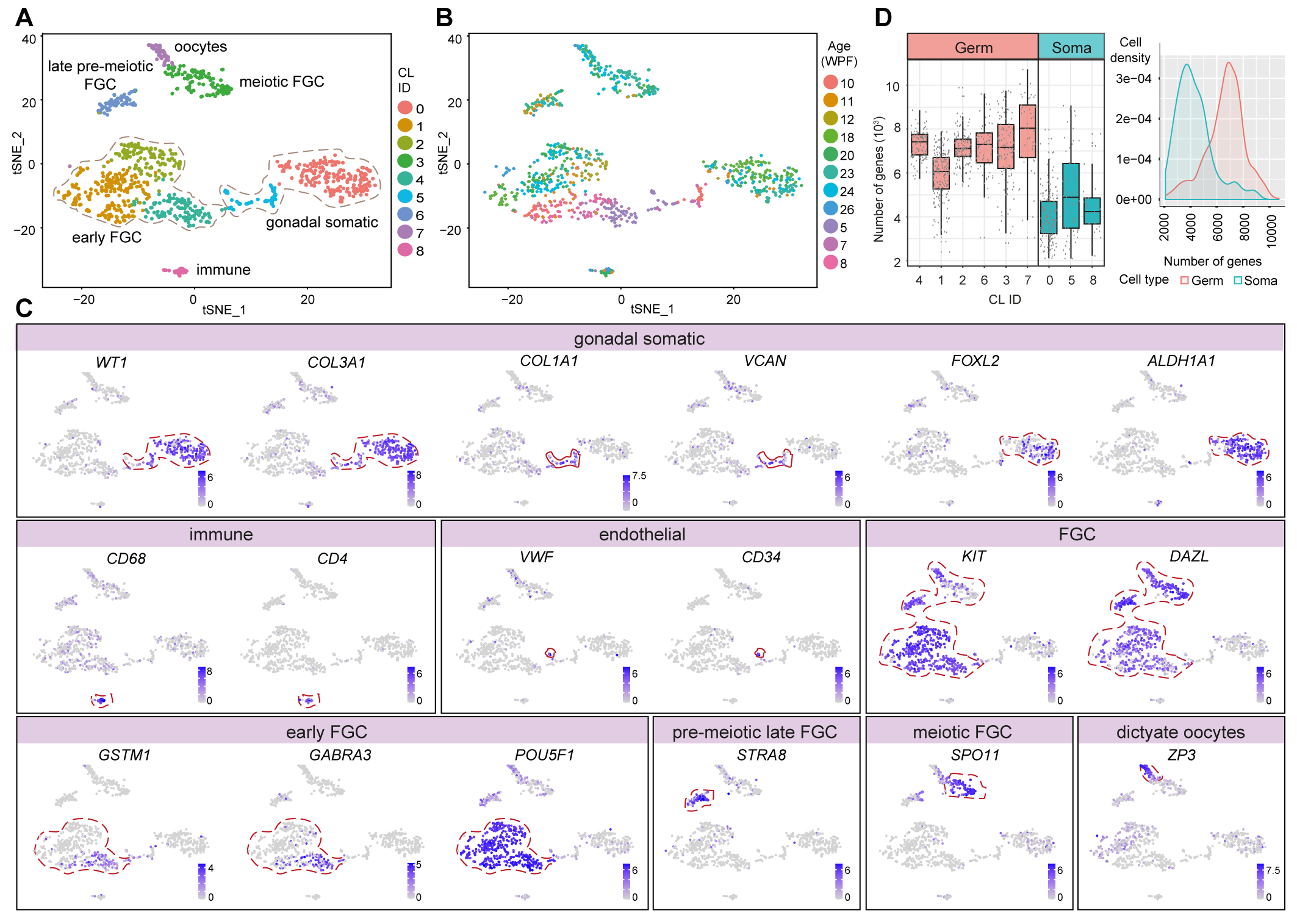

Supplement: S1 Fig — (A-C) tSNE plots showing cell cluster identity (CL ID) for female fetal gonadal cells (FGC, fetal germ cells) (A), age in weeks post-fertilization (WPF) (B) and expression of markers for each major cell type (C). (D) Box plot and cell density graph showing numbers of genes expressed per cell in the germline and somatic cells in the female gonads per cluster. (TIF) [file pgen.1009773.s001.tif]

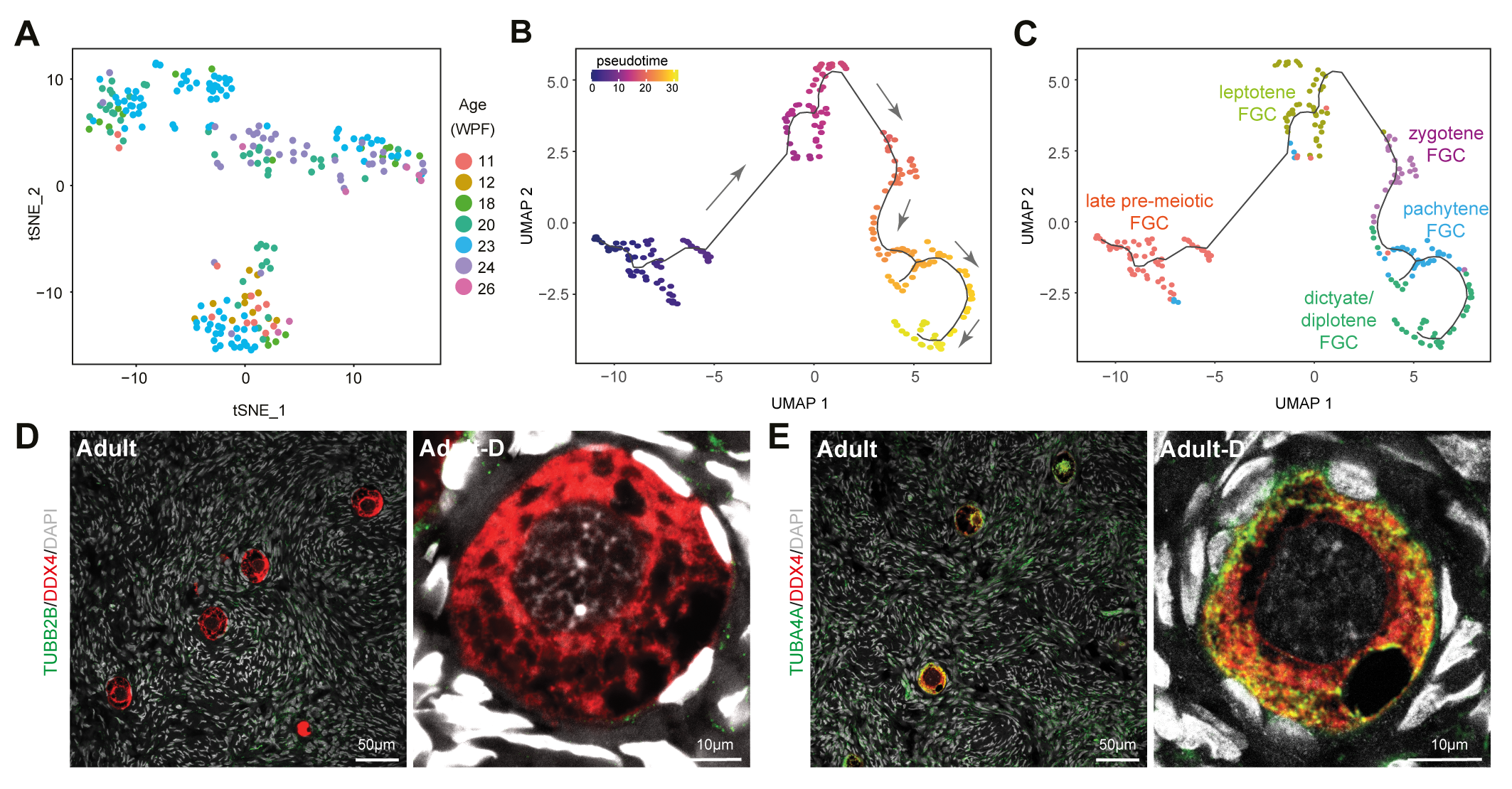

Supplement: S2 Fig — (A) tSNE plot of female FGC in pre-meiotic and meiotic stages coloured by age in weeks post-fertilization (WPF). (B-C) Pseudotime analysis of female pre-meiotic and meiotic FGC by Monocle 3. UMAP plots show pseudotime (B) and cluster identification (C). (D) Immunofluorescence for TUBB2B and DDX4 in primordial follicles with oocytes arrested in diplotene in adult ovary. Scale bars are 50μm in the overview image (left) and 10μm in the high magnification image (right). (E) Immunofluorescence for TUBA4A and DDX4 in primordial follicles with oocytes arrested in diplotene in adult ovary. Scale bars are 50μm in the overview image (left) and 10μm in the high magnification image (right). (TIF) [file pgen.1009773.s002.tif]

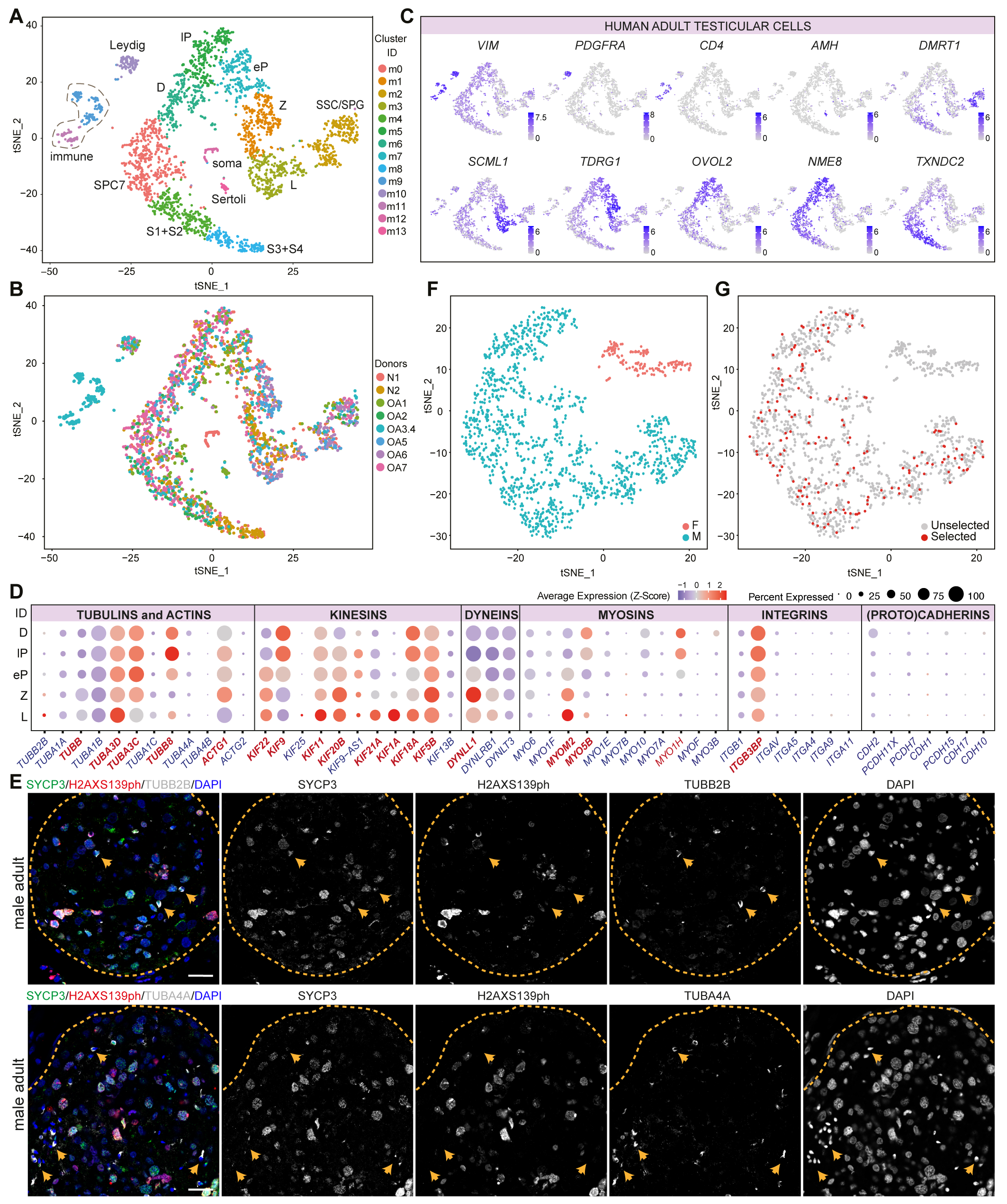

Supplement: S3 Fig — (A-C) tSNE plots showing cell cluster identity (CL ID) representing the main cell types (A), individual donors (B) and expression of known markers for each major cell population (C). (SSC/SPG, spermatogonial stem cells/spermatogonia; L, leptotene; Z, zygotene; eP, early pachytene; lP, late pachytene; D, diplotene; SPC7, spermatocyte 7; S1/S2/S3/S4, four stages of spermatids). (D) Dot plot showing scaled average expression (Z-score) of cytoskeleton, motor and cell adhesion gene families in different meiotic prophase I stages. Gene names in red are mDEGs, in red and bold are also fDEGs and in blue are not mDEGs. (E) Immunofluorescence of TUBB2B, SYCP3 and H2AXS139ph (top) and TUBA4A, SYCP3 and H2AXS139ph (bottom) in adult testes. Orange arrows indicate TUBB2B or TUBA4A positive spermatids, orange dashed lines mark the seminiferous tubules. Scale bars are 20μm. (F-G) tSNE plots displaying female and male cells in L, Z, P and D, coloured by sex (F) and showing the selected male cells used in further analysis (G). (TIF) [file pgen.1009773.s003.tif]

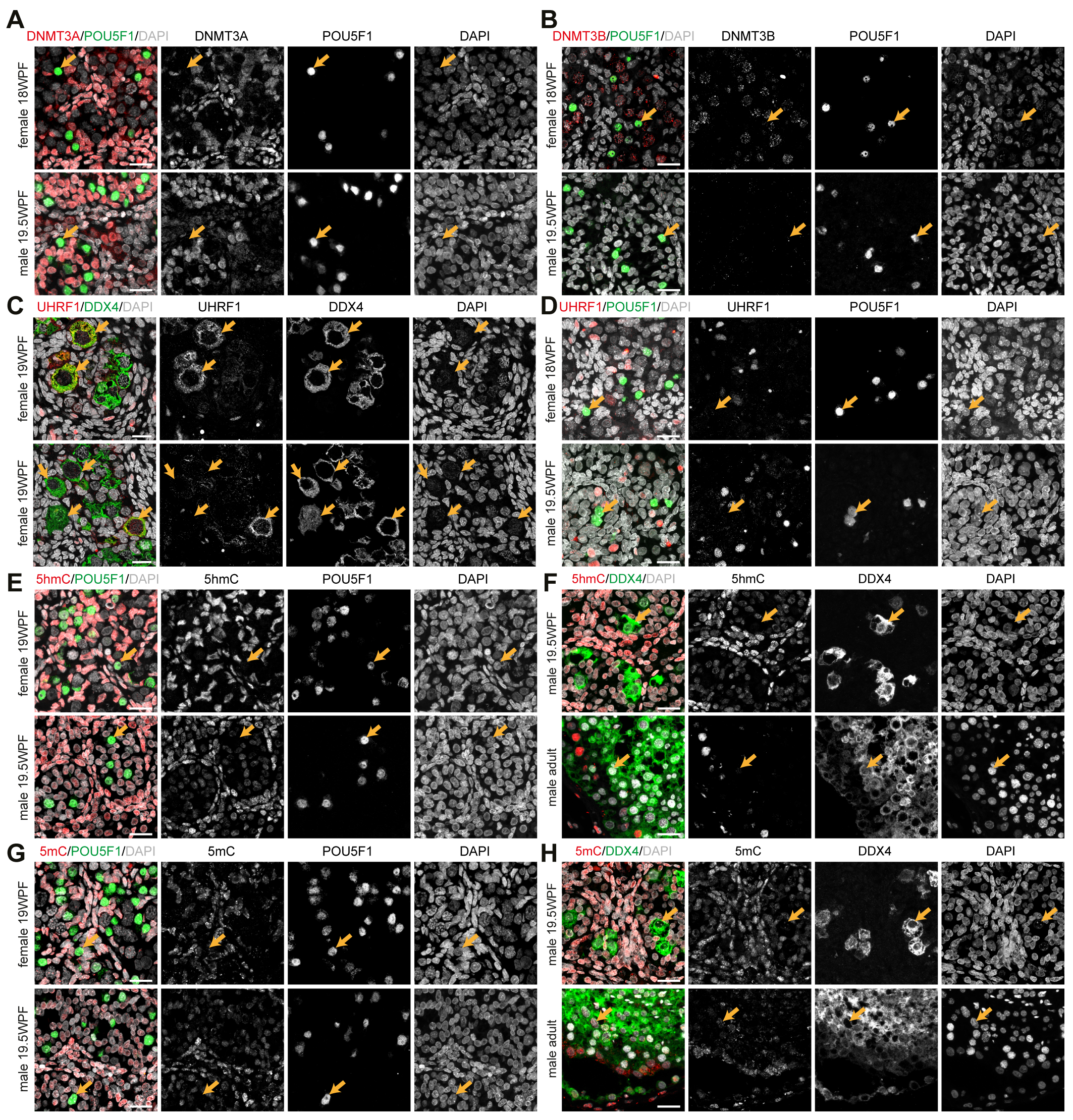

Supplement: S4 Fig — (A-B) Immunofluorescence for DNMT3A (A) and DNMT3B (B) in POU5F1+ FGC in second trimester ovaries and testes. Orange arrows indicate representative POU5F1+ FGC. Scale bars are 20μm. (C-D) Immunofluorescence for UHRF1 in DDX4+ FGC (C) and POU5F1+ FGC (D) in second trimester ovaries and testes. Orange arrows indicate representative FGC. Scale bars are 20μm. (E) Immunofluorescence for 5hmC in POU5F1+ FGC in second trimester ovaries and testes. Orange arrows indicate representative POU5F1+ FGC. Scale bars are 20μm. (F) Immunofluorescence of 5hmC in DDX4+ FGC from second trimester and adult testes. Orange arrows indicate representative DDX4+ FGC. Scale bars are 20μm. (G) Immunofluorescence for 5mC in POU5F1+ FGC in second trimester ovaries and testes. Orange arrows indicate representative POU5F1+ FGC. Scale bars are 20μm. (H) Immunofluorescence of 5mC in DDX4+ FGC from second trimester and adult testes. Orange arrows indicate representative DDX4+ FGC. Scale bars are 20μm. (TIF) [file pgen.1009773.s004.tif]

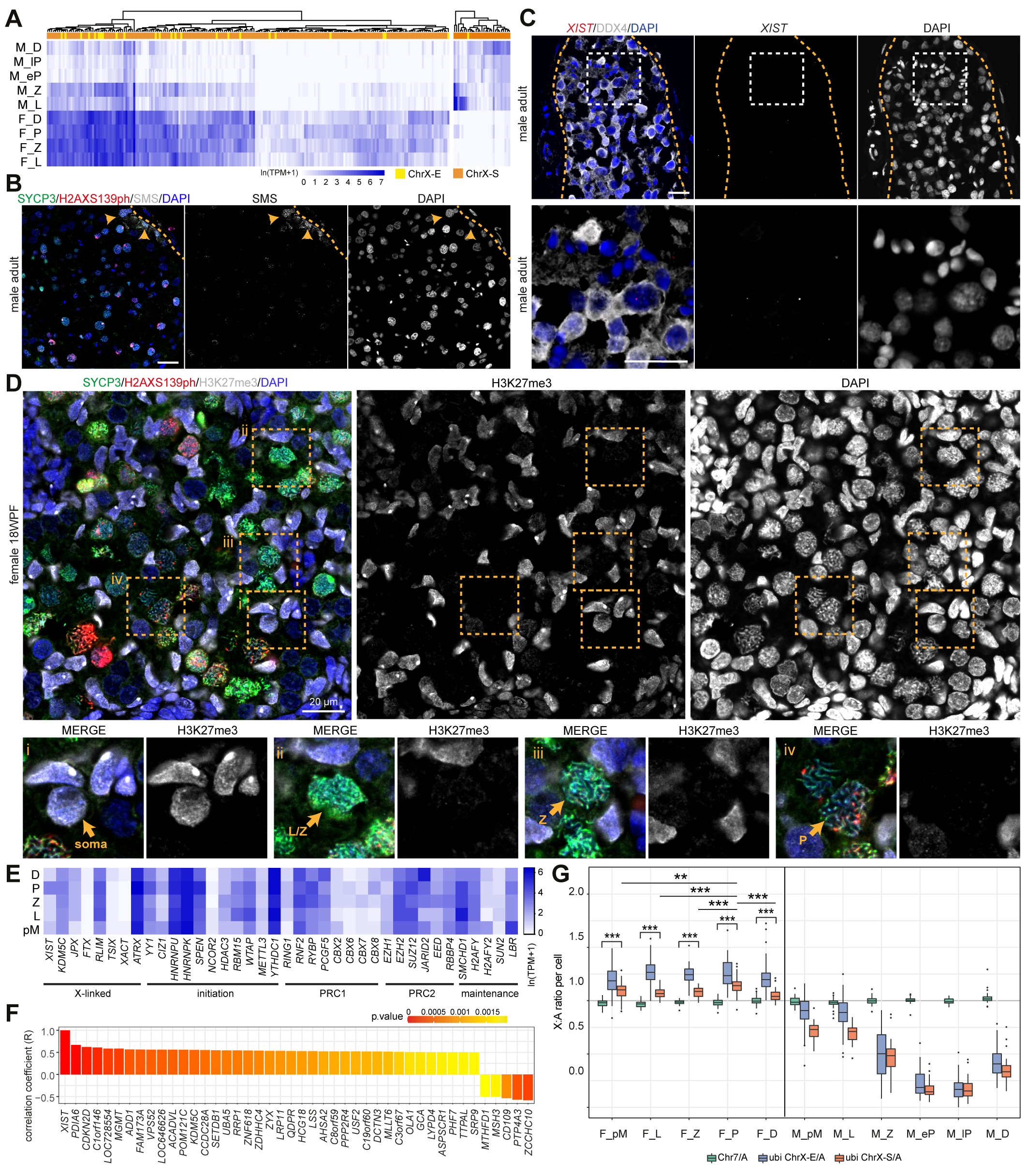

Supplement: S5 Fig — (A) Heatmap of X-linked sex-enriched genes (ChrX-Escape and ChrX-Subject to XCI) in male and female germ cells during meiotic prophase I. (B) Immunofluorescence for SMS, SYCP3 and H2AXS139ph in adult testes. Orange arrowheads indicate SMS in spermatogonia, orange dashed lines mark the border of seminiferous tubule. Scale bar is 20μm. (C) RNA FISH for XIST combined with immunofluorescence for DDX4 in adult testes. White dashed box is shown in high magnification (bottom). Orange dashed lines mark the border of seminiferous tubule. Scale bars are 20μm. (D) Immunofluorescence for H3K27me3, SYCP3 and H2AXS139ph in 18WPF ovaries. Orange dashed boxes indicate the areas shown in high magnification. Orange arrows indicate somatic cells or FGCs in their meiotic prophase I stages. (soma, somatic cells; L/Z, late leptotene-early zygotene; Z, zygotene; P, pachytene). Scale bar is 20μm. (E) Heatmap showing expression of XIST and XCI related genes in female FGC during meiotic prophase I (pre-meiotic, pM; leptotene, L; zygotene, Z; pachytene, P and diplotene/dictyate, D). (F) Bar chart showing genes that showed correlation with XIST expression (Pearson correlation coefficient R>0.5 or R<-0.5) in female pachytene FGC. Colour key is scaled by adjusted P-value. (G) X:A ratio of female and male germ cells in different stages. Box plot shows the mean expression ratios of ubiquitously expressed genes (ubi) from Chr7, ChrX-E or ChrX-S to all ubiquitously expressed genes from autosomes per cell. Cells from M-pL were actually cells from male spermatogonial stem cells/spermatogonia cluster (mCL2). Statistical significance was assessed using Wilcoxon rank-sum test or Wilcoxon signed-rank test for female germ cells, **P < 0.01, ***P < 0.001. (TIF) [file pgen.1009773.s005.tif]
